# Faster and more precise isotopic water analysis of discrete samples by predicting the repetitions’ asymptote instead of averaging last values

**DOI:** 10.1016/j.mex.2022.101656

**Published:** 2022-03-03

**Authors:** Nico Hachgenei, Véronique Vaury, Guillaume Nord, Lorenzo Spadini, Céline Duwig

**Affiliations:** a IGE, Univ. Grenoble Alpes, CNRS, IRD, Grenoble INP, Grenoble, France; b iEES, Sorbonne Univ., Paris, France

**Keywords:** Picarro, Water stable isotopes, Cavity ring-down spectroscopy (CRDS), Calibration, Hydrology, Tracer, aol, average of the last injections, exp, method fitting an exponential function $y=a\cdot e^{-b\cdot x}+c$ to repeated injections, inv, method fitting $y=\frac{a}{x}+b$ to repeated injections

## Abstract

Water stable isotope analysis using Cavity Ring-Down Spectroscopy (CRDS) has a strong between-sample memory effect. The classic approach to correct this memory effect is to inject the sample at least 6 times and ignore the first two to three injections. The average of the remaining injections is then used as measured value. This is in many cases insufficient to completely compensate the memory effect. We propose a simple approach to correct this memory effect by predicting the asymptote of consecutive repeated injections instead of averaging over them. The asymptote is predicted by fitting a $y=\frac{a}{x}+b$ relation to the sample repetitions and keeping b as measured value. This allows to save analysis time by doing less injections while gaining precision. We provide a Python program applying this method and describe the steps necessary to implement this method in any other programming language. We also show validation data comparing this method to the classical method of averaging over the last couple of injections. The validation suggests a gain in time of a factor two while gaining in precision at the same time. The method does not have any specific requirements for the order of analysis and can therefore also be applied to an existing set of analyzes in retrospect.

• We fit a simple $y=\frac{a}{x}+b$ relation to the sample repetitions of Picarro L2130-i isotopic water analyzer, in order to keep the asymptote (b) as measured value instead of using the average over the last couple of measurements.
• This allows a higher precision in the measured value with less repetitions of the injection saving precious time during analysis.
• We provide a sample code using Python, but generally this method is easy to implement in any automated data treatment protocol.

## Graphical abstract

**Figure:**
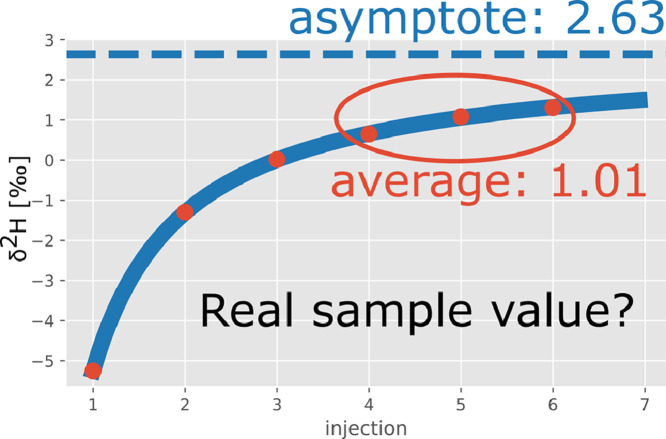

Specifications table

**Table:** 

Subject Area:	
More specific subject area:	Environmental tracers
Method name:	Asymptotic Approximation Calibration in Cavity Ring-Down Spectroscopy (CRDS)
Name and reference of original method:	Picarro L2130-i manual [3]
Resource availability:	A Python program applying this method is provided in the Supplementary Material, as well as a validation dataset

## Introduction

Cavity Ring-Down Spectroscopy (CRDS) such as the *Picarro L2130-i Isotopic Water Analyzer* is an inexpensive, easy to use and relatively precise method for the analysis of stable isotope ratios of the water molecule. However, this method is subject to a strong between-sample memory effect (or carryover) as well as drift with time [1,5,8]. The memory-effect is caused by residual water of previous samples inside the sampling syringe, transfer path or the cavity itself [2,8]. In their *L2130-i Isotopic Water Analyzer* manual [3], Picarro states that in order to ‘completely eliminate memory effects’, each sample should be injected 6 times (p. I-14) and the first two to three values should be ignored. Then the average of the last couple of injections is retained as measured value. This method will be referred to as aol (average of last samples) in the following. Laboratories using CRDS like the *Picarro L2130-i Isotopic Water Analyzer* often either use this approach, sometimes with a higher number of injections if precision is of importance, or rely on custom programs to try and correct for the memory effect. When analyzing contrasted samples (e.g., the standards or contrasted environmental samples like rain water), a clear, monotonous trend may still be visible in the repetitions, even beyond 10 injections [1,2]. Vallet-Coulomb et al. [5] detected a memory effect after up to 45 injections of the same sample.

There have been other publications with different approaches to try and correct the memory effect via software solutions, sometimes combined with the analysis of a particular sequence of known standards. These approaches have been grouped differently by different authors (e.g., [4,6]). The simplest solution is averaging over the last couple of injections and ignoring the first injections (aol). This assumes that enough injections were performed for the value to stabilize. As mentioned above, many injections are often necessary to reach stabilization of measured values. This method is simple to apply and therefore still largely used and recommended by Picarro, even though it requires many injections making it money- and time-consuming and in reality stabilization is often not reached, creating a bias in the result. More advanced approaches include the application of correction coefficients that quantify the memory effect as a function of the difference between the current and previous sample [1,6,8]. These correction coefficients correspond to the proportion of residual water vapor from the previous sample. Wassenaar et al. [8] propose a particular order of analysis and a software (Lims for Lasers) which allow to quantify the memory effect in between two samples by comparing the result of a standard analyzed after a contrasting standard to the result of a repeated analysis of the same standard. They use the average of non-ignored injections as only information for each sample. Gröning [1] proposes an excel-based software-tool estimating the memory effect for each injection of the sample analysis by estimating the effect of the previous sample on each injection of the following sample for each pair of two consecutive samples. Van Geldern & Barth [6] use a similar approach, estimating memory coefficients from a series of known standards and update these memory coefficients using daily repetition of the series of standards. Guidotti et al. [2] argue that there are multiple pools of residual water of different size that are mixed with the sample over different periods of time. Therefore, they propose to use one exponential model for each pool's contribution to the overall memory effect. This method has a solid physical base in the assumption of different pools, the isotopic signature of which changes exponentially with subsequent injections. The model parametrization remains empirical as the pools are not physically defined and the parameters are calibrated from repeated analyzes of known standards. The most rapid pool would change its signature to the new sample's signature almost instantly with the first injection while the signature of the slowest pool would remain far from the samples signature even over many consecutive injections. Therefore, their memory correction algorithm considers not only the previous sample but all previously injected samples since the start of the series. This is a very complete and physically sound approach however requiring prior assumptions on the number of pools contributing to the memory effect as well as the calibration of two parameters per pool in addition to the initial conditions (initial isotopic signature of each pool).

Vallet-Coulomb et al. [5] perform a detailed comparison of different memory correction methods on the same sequence of standards and samples treating known standards as unknown samples with ten injections each. Firstly, they apply an aol method by ignoring the first six samples and averaging over the remaining four. Secondly, they use one method where for each sample the residual vapor has the signature of the previous sample and its contribution decreases with the number of injections. This resembles the methods used by Gröning [1] and Van Geldern & Barth [6]. Thirdly they use the method presented by Guidotti et al. [2] using two residual water pools. Finally, they developed a simplified version of the method proposed by Guidotti et al. [2], where the exchange between the sample and the residual vapor is constant, but the signature of the residual vapor changes with every injection and depends on the four previous samples. Instead of assuming the number of contributing pools and calibrating parameters of multiple exponential functions to mathematically approximate the evolution of the isotopic signature of each pool, they use measured memory correction coefficients obtained beforehand from subsequent series of 50 injections of known standards. The aol method overestimated the sample values as they were preceded by a high sample. The method from Guidotti et al. [2] also overestimated the sample values, which the authors explain with the method being developed for extremely contrasted artificially spiked samples and not adapted for environmental waters. The method only depending on the previous sample's value overcorrected the result and the method developed by the authors produced the best results with a slight overcorrection.

We propose a simple approach to correct the memory effect that can be implemented in any programming language that is used for data treatment and we provide a ready-to-use Python implementation together with the validation data. This approach works in a different way than those developed by others that are presented above. Instead of estimating the memory effect from previous samples and then correcting the value of each injection for it, we consider each sample individually and predict the asymptote of repeated injections from the trend observed through the injections. The application of this approach does not require profound knowledge of the analytical system and is hardly more complex than averaging over the last samples but adds precision and requires less injections per sample.

In addition to the memory effect, the instrument's drift over time needs to be corrected for. In contrast to the memory effect, this correction is relatively straight forward and does not differ much between the different studies. It is estimated by regularly measuring known standards and quantifying their trend over time and can then be corrected through linear approximation. A simple drift correction is also briefly described at the end of the methods section and included in the Python program provided with this article.

## Method details

In this section, the principle of the proposed memory correction method is introduced, including a step-by-step description. Thereafter, the whole workflow of the provided calibration program is presented briefly including the calibration and drift correction. This workflow was applied to three Picarro standards and a few natural stream water samples. The stream water samples will not be further presented in this article. In the following validation section, different tests were performed to compare the proposed method to the use of the classical aol method as well as an exponential approximation.

Assuming the asymptote of the measured value per injection to be the ‘ideal’ measured value (i.e. unaffected by the memory effect), an average over the last measured values is biased toward the precedent sample as long as the data present a trend. Therefore, we aimed to directly estimate the asymptote from the trend in the repeated injections. This method uses the information from all injections in order to predict the asymptote instead of eliminating this valuable information and only using the last measured values. It does not have particular requirements on the order of analysis and can deal with variable number of injections (e.g. if more injections are used for the standards than for the samples). Different asymptotic functions may be used to try and reproduce this trend. We found $y=\frac{a}{x}+b$ (called inv hereafter) to give good results. y is the measured isotope ratio, x is the number of injection, a and b are parameters that are optimized to fit the data points, where a is a scale factor and b is the asymptote. Fitting an exponential function $y=a\cdot e^{-b\cdot x}+c$ (called exp hereafter) did not significantly improve the calibration compared to the aol method (see method validation section for details).

The procedure of the inv method is to optimize a and b to obtain a good fit for measured value vs. injection number for each of the analyzed samples. We use Python's scipy module and its optimize.curve_fit function [7]. The program is provided in the supplementary material.

This method is applied to any analyzed sample (i.e. standards and unknown samples). The memory correction can be combined with any calibration workflow. See the end of this section for details on the full workflow of the provided Python program.

No matter which programming language is used, we strongly recommend either fitting a linear function to 1/x-transformed data or using increasing weight/decreasing uncertainty with number of injection when fitting directly on the data-scale. If all points are equally weighted, the first injections have a stronger influence on the fit as they are further away from the mean and thus weigh stronger in the fitting algorithms cost function. We obtain this weighting in scipy.optimize.curve_fit through the sigma argument, which describes the uncertainty of each data point and corresponds to the inverse of each point's weight. The cost function is $chisq=\sum {\left(\frac{y-f(x)}{sigma}\right)}^{2}$. Sigma should thus decrease with number of injection in order to increase the weight. We used $sigma\left(x\right)=\frac{1}{1+0.5\cdot x}$ in order to have a weight of 1+0.5·x for the x^th^ injection. Varying this parameter slightly (e.g. using $sigma\left(x\right)=\frac{1}{x}$ or sigma(x)=exp(−0.2x)) did not significantly impact the results presented in the method validation section.

We also recommend to verify the existence of a trend in the measured values vs. injection number (and thus presence of a memory effect) before fitting the inv function. If the data does not present a trend (too little difference between first and last value) which happens, if the sample is very close to the previous sample and the memory effect does not affect the measured value, we use the classic method of averaging over the last injections. A minimum difference between first and last value of 0.15 for δ^18^O and 0.4 for δ^2^H worked well in our case. If no satisfying fit can be found (e.g. there is a difference between start and end, but due to noise with no clear trend), again, the aol method should be used. In our program, the curve_fit function produces a runtime error if no fit can be found and the aol method is used. It is recommended to plot each series of injections with the fit in order to visually verify the fit by scrolling through the figures.

The procedure of memory correction can be summarized with the following steps to be applied to each analyzed sample, one by one.

1. Verify if the difference between first and last value is larger than a threshold, if so continue to step 2, else do aol.
2. Try to fit a $y=\frac{a}{x}+b$ function to measured value vs. injection number by optimizing a and b to minimize the cost function $chisq=\sum {\left(\frac{y-f(x)}{sigma}\right)}^{2}$, where sigma prevents overfitting to the first injections. If this fails use aol, else continue to step 3.
3. Retain b as the measured value of the sample.

Given that each injection in high precision mode takes about 8 min and that inv seems to yield a higher precision after only six injections compared to aol after 12 injections (see method validation section), analysis could be sped up a factor two using this method while gaining in precision. Taking into account the necessity of regularly reanalyzing the standards due to possible drift of the device, the time gain is increased as more samples may be analyzed in between two repetitions of the standards.

The step-by-step calibration workflow of the provided Python program is as follows:

1. For each set of standards (in our example data there is one in the beginning and one in the end).
  a. Inv method is applied to estimate each standard's measured value.
  b. A calibration is performed by fitting a linear relation to real vs. measured values of the standards.
  c. The two fitted parameters (intersection and slope) are saved together with the central time of analysis of this set of standards (i.e. the middle time between first injection of the first standard and last injection of the last standard).
2. The calibration parameters (interception and slope) are interpolated to each sample's central measurement time (i.e. the middle time between first and last injection), using the two neighboring sets of standards in order to obtain one individual set of calibration parameters for each sample that is corrected for the instrument's drift. The parameters are interpolated along the time-axis, assuming that instrument drift is linear with time for the duration between each two sets of standards.
3. The measured value of each sample is calculated by applying the inv method to its repeated injections.
4. The predicted isotopic ratio of each sample is calculated by applying its individual calibration parameters to the measured value.

This workflow is applied to δ^18^O and δ^2^H in parallel independently. The program is written in Python 3 and tested on version 3.8, but should be compatible with near future versions.

## Method validation

For validation, we compare the method of fitting an asymptotic function to the method officially recommended by Picarro (aol). We used 3 different standards: PICARRO ZERO, MID and DEPL. They were stored in sealed glass ampoules, opened on the day of analysis and directly transferred into analysis vials. Their concentrations and uncertainties are given in Table 1. We also analyzed IAEA VSMOW2 (Vienna Standard Mean Ocean Water) and SLAP2 (Standard Light Antarctic Precipitation) (see supplementary material), however they were not used for calibration or verification as they had been opened beforehand and stored in a recipient containing air. We did 12 injections of each standard in high precision mode (8 min per injection). The order of analysis was as follows.

1. Tap water
2. PICARRO DEPL
3. PICARRO MID
4. Tap water
5. PICARRO ZERO

**Table 1:** Values and uncertainties of the standards used. (Picarro Lot #0517–12–06.x, Certificate C0350).

Name	δ^18^O [‰]	Uncertainty	δ^2^H [‰]	Uncertainty
PICARRO ZERO	0.3	±0.2	1.8	±0.9
PICARRO MID	−20.6	±0.2	−159.0	±1.3
PICARRO DEPL	−29.6	±0.2	−235.0	±1.8

In order to estimate the number of injections necessary to obtain acceptable precision, we dropped the results from the last injections and repeated the calibration on this reduced data in order to simulate an analysis using less injections. This way we could compare the results of doing 2, 3, 4, 5, 6, 7, 8, 9, 10, 11 and 12 injections. For the aol we chose to average over the last 5, 4, 3, 2, 1 injections when there were at least 9, 7, 5, 3, 2 injections respectively in total.

**Fig. 1:**
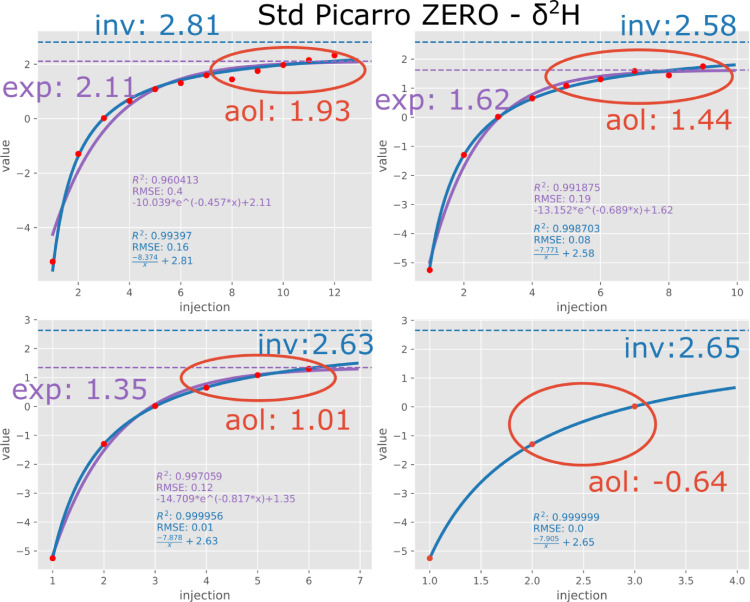
Application of aol, inv and exp to δ^2^H of Picarro ZERO standard after 12, 9, 6 and 3 injections respectively. Red dots are measurements, the blue curves are inv function fits and the purple curves are exp function fits. Dashed lines are corresponding asymptotes.

Fig. 1 illustrates the application of aol and inv for different numbers of injections (uncalibrated raw measurement). The result of inv does not vary much (between 2.58 and 2.81 for this standard), while aol varies much more (from −0.64 to +1.93). We also note that even after 12 injections, the ranges of values obtained by the two methods do not overlap. This can be explained by the trend that still persists after 12 injections, indicating that the measurements have not yet reached their asymptote. The average remains thus biased towards the previously measured sample. The exp method generally results in values close to aol. It either fits the first couple of injections or the last ones but it fails to reproduce the overall shape. This is in accordance with Guidotti et al. [2] who state that there are multiple residual water pools with different exponentially evolving signatures. The combined effect of these pools can therefore not be covered by one single exponential function. The inv function however seems to be able to match the combined effect of these different pools.

After extracting each standard's measured value with the different methods, a calibration is performed over the three standards by fitting a linear relation between measured and real values. In order to estimate the improvement from using an asymptotic estimation instead of averaging over the last couple of values, we calculated the coefficient of determination R² of this calibration function. According to the manual of the *Picarro L2130-i* [3], the relationship between the machines signal and the real isotopic ratio of the sample is perfectly linear. Therefore, a higher R² of the calibration indicates a more precise estimation of each standard's measured value on the scale of the instruments raw estimation.

**Fig. 2:**
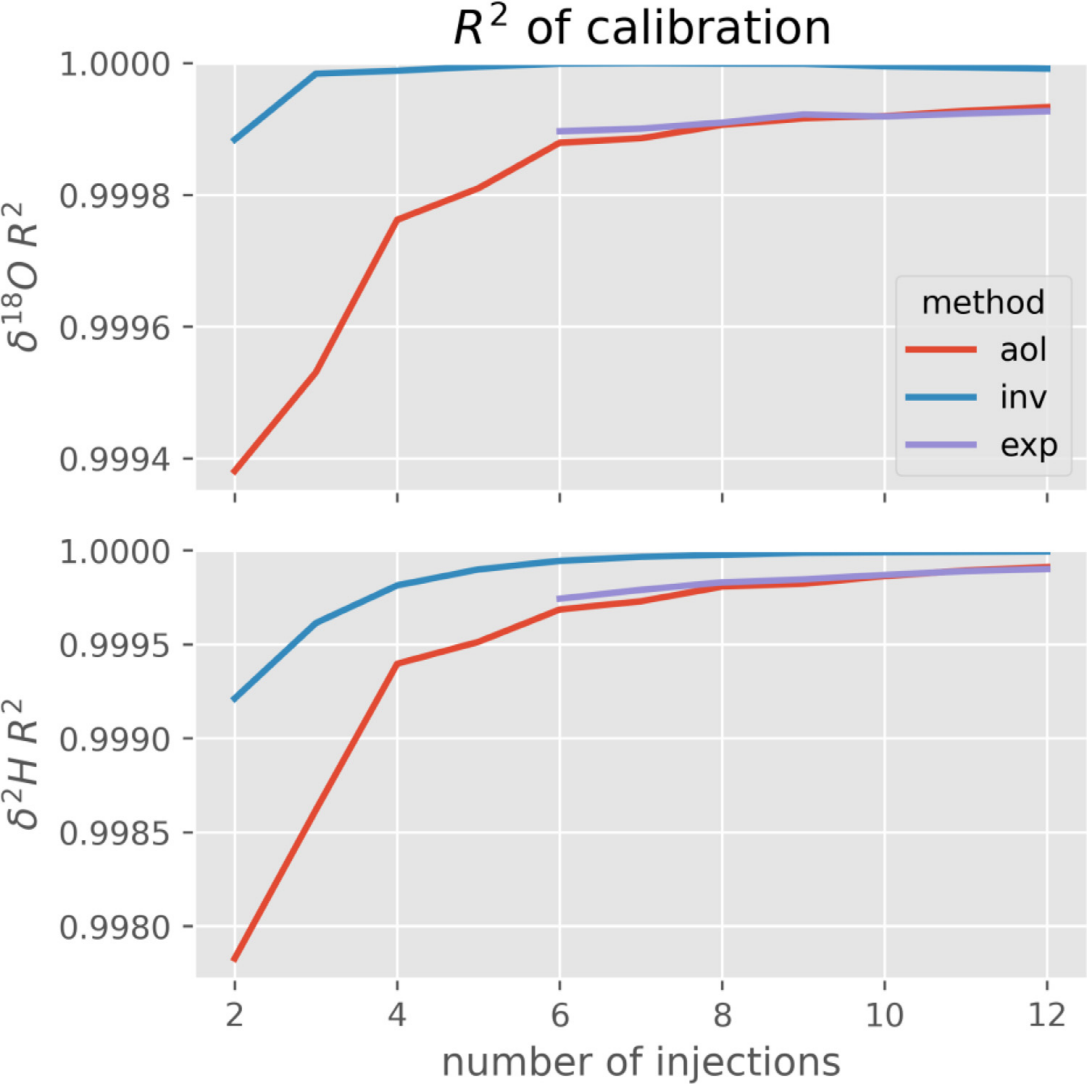
Goodness of fit (R²) of the calibration curve obtained from the three standards for the three presented methods as a function of the number of injections.

A calibration was performed for each method and each number of injections Fig. 2. shows the R² for δ^18^O and δ^2^H as a function of the total number of injections for the three tested methods (aol, inv, exp). It can be seen that R² improves with the number of injections for all of the methods. R² was higher when applying inv method to estimate the measured value from repeated injections, indicating a more linear relation between measured and real values with less noise and thus a better calibration. The lower R² of the calibration based on the aol method can be explained by the memory effect causing an overestimation of PICARRO DEPL and underestimation of PICARRO MID & ZERO leading to a less linear relation between measured and real values. Exp produces very similar results as aol, while inv produces a good fit even with very few injections. As little as four injections seem to produce acceptable results, however we strongly recommend to do at least six injections. With only six injections, the calibration fit using inv method is better than that using aol method with twelve injections. This indicates, that the approximation method removes a significant part of the bias in measurement caused by the previous sample through memory effect.

**Fig. 3:**
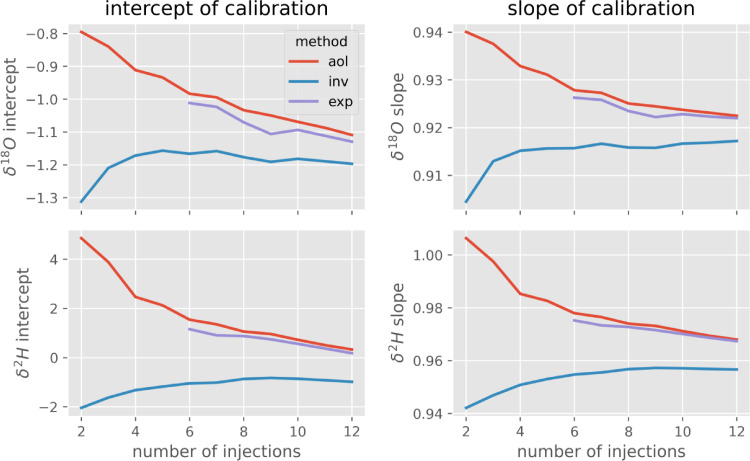
Effect of the number of injections on the two parameters of the calibration function (slope and intercept) for the three methods.

Fig. 3 shows the two parameters (slope and intercept) of the retained calibration (real standard value = f (measured value)). For aol, the effect of using too few injections is a higher slope and a higher intercept (for both isotopes). This translates to a lower maximum and higher minimum measured value, indicating that the ‘extreme’ standards are ‘pulled’ toward the middle (or toward the previously measured value). This trend continues all the way to our maximum of 12 injections, indicating a potential of improvement even beyond 12 injections.

The exp method remains close to the aol method. This is because an exponential function that well represents the strong difference between the first injections too rapidly approaches zero thereafter. Thus, the exponential function tends to have its asymptote somewhere between the last couple of injections without extrapolating their trend into the future. The calibration results of the inv method are much less sensitive to the number of injections. The inv method produces relatively constant intersection and slope after four (δ^18^O) to six (δ^2^H) injections.

Fitting an exponential function would correspond to the hypothesis of one residual water pool with a first order mixing with water from the new sample, diluting it by the same factor with each new injection. This hypothesis can be rejected, as the exponential function underestimates the projected memory effect. In reality, the memory effect has a longer ‘tail’ (i.e. seems to be more time-persistent) than a first order dilution. As mentioned above, this may be explained by the existence of multiple exponential pools, the combined effect of which cannot be reproduced by one single exponential relation [2]. This longer ‘tail’ can be reproduced by the inv method.

**Fig. 4:**
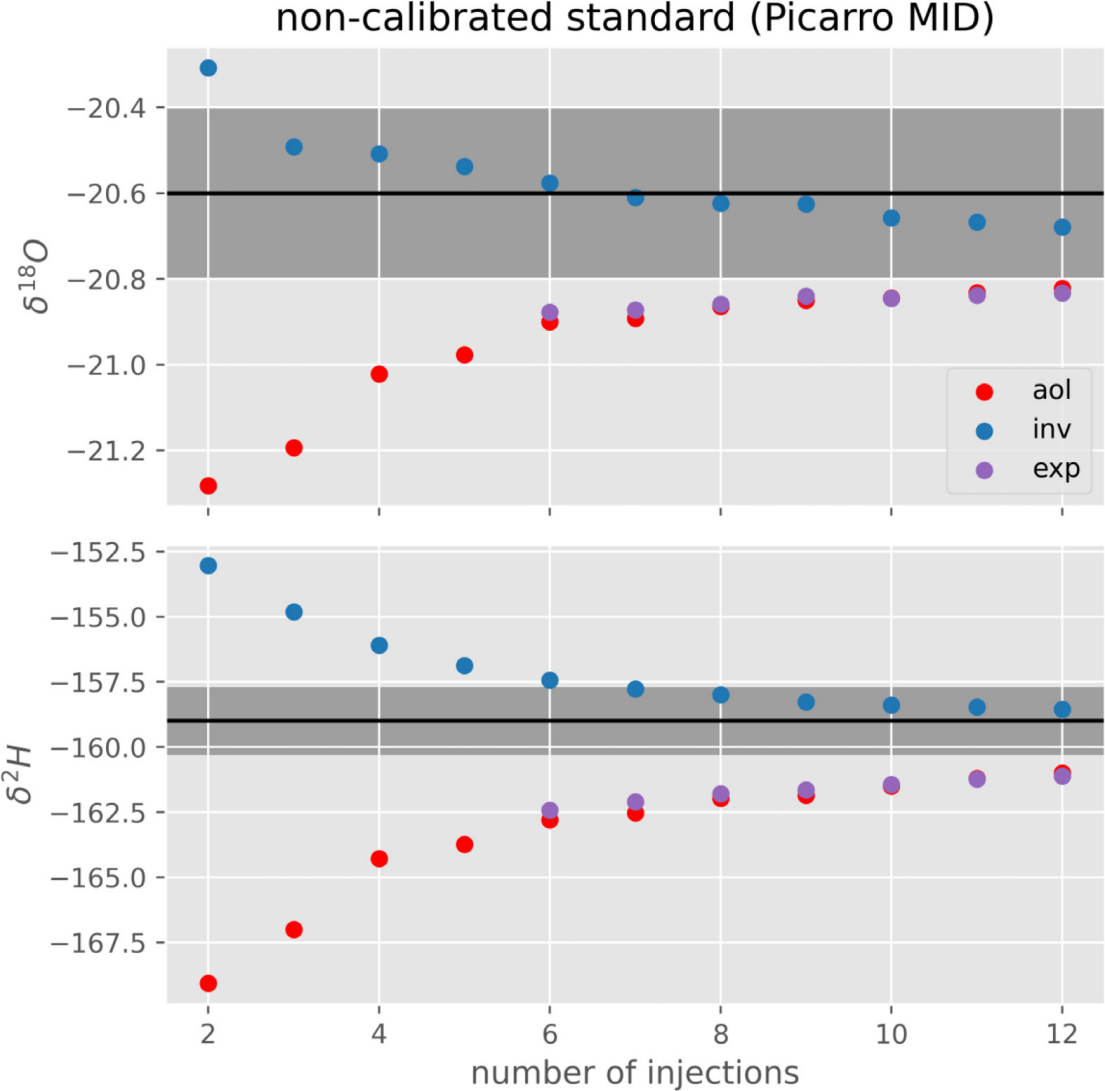
Prediction of the intermediate standard, that for this example has not been used for calibration. Measured using the three methods calibrated on the two remaining standards (DEPL and ZERO). The black line represents the actual value of the standard. The shaded area corresponds to the uncertainty of the standard as given by Picarro. The points are prediction from the three different methods calibrated on x injections and measuring the sample with the same number of injections.

As an additional validation of the method we performed a calibration on the two extreme standards only (PICARRO DEPL and ZERO) and predicted the concentration of the intermediate standard (PICARRO MID) using this calibration. We did this for 2 to 12 injections again to verify the potential of the method to reduce the number of injections necessary in order to obtain acceptable results Fig. 4. shows the predicted value of PICARRO MID as a function of the number of injections for the three methods. The aol and exp methods do not reach within one standard deviation of the real value even after 12 injections for both isotopes. The inv method gets within uncertainty bounds with 3 injections (δ^18^O) or 7 injections (δ^2^H).

## Declaration of Competing Interest

The authors declare that they have no known competing financial interests or personal relationships that could have appeared to influence the work reported in this paper.

## Appendix Supplementary materials

Image, application 1
